# Repurposing ALK inhibitors as influenza and corona virus antivirals targeting lymphocyte tyrosine kinase (LTK)

**DOI:** 10.1016/j.virusres.2026.199759

**Published:** 2026-06-09

**Authors:** Elias Tjärnhage, Thea Kristin Våtsveen, Melinda Raki, David Nemazee, Hesso Farhan, Ludvig Munthe, Gunnveig Grodeland

**Affiliations:** a Institute of Clinical Medicine, University of Oslo, Oslo, Norway; b Department of Immunology, Oslo University Hospital, Oslo, Norway; c KG Jebsen Centre for B cell malignancy, Institute of Clinical medicine, University of Oslo, Oslo, Norway; d PRIMA, Precision Immunotherapy Alliance, University of Oslo, Oslo, Norway; e Department of Pathology, Oslo University Hospital, Oslo, Norway; f The Scripps Research Institute, La Jolla, CA, USA; g Institute of Basic Medical Sciences, University of Oslo, Oslo, Norway; h Institute of Pathophysiology, Medical University of Innsbruck, Innsbruck, Austria; i Centre for Pandemics and One-Health Research, Institute of Health and Society, University of Oslo, Norway

## Abstract

The development of effective antiviral drugs for pandemic scenarios is a significant challenge, particularly due to the rapid emergence of drug-resistant viral strains and a paucity of drugs that have broad efficacy across viral families. A promising strategy is so called host-targeted therapeutics which inhibit host-mechanisms that viruses rely on instead of viruses themselves. LTK, an endoplasmic reticulum-resident kinase, plays a crucial role in ER-to-Golgi trafficking, a process exploited by many viruses during their life cycle, and is therefore a potential candidate for host-targeted antiviral therapeutics. LTK is highly homologous to ALK, a tyrosine kinase that can be aberrantly expressed by cancer cells, and importantly, LTK, but not ALK, is expressed in the lung and small intestine. We here repurposed ALK-inhibitors that also inhibit LTK, and investigated if this inhibition could reduce viral load and serve as a new class of antiviral drug. *In vitro*, influenza and SARS-CoV-2 viruses could be inhibited by the LTK inhibitors ceritinib, crizotinib, entrectinib, ensartinib, brigatinib, and alectinib, but not lorlatinib. Importantly, the repurposed ALK-inhibitors crizotinib, brigatinib, and ceritinib also gave some protection in mice against lethal viral challenges with influenza and SARS-CoV-2, respectively. The study highlights the potential of LTK inhibition as target for a new class of host-targeted antivirals therapeutics.

## Highlights

• Antiviral therapies are an important first line of defense against new viruses.
• Host-targeted antivirals can circumvent escape mutations in variable viruses.
• We evaluate repurposed cancer drugs for protection against influenza and SARS-CoV-2.
• Inhibition of protein secretion reduced viral infection in cell cultures and mice.

## Graphical abstract

**Figure:**
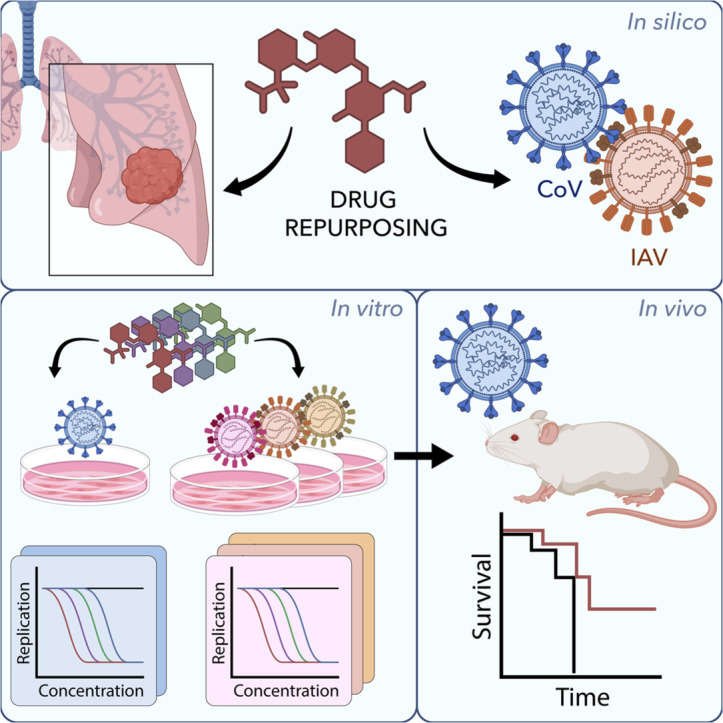

Rapidly evolving respiratory viruses like influenza and coronaviruses cause substantial morbidity in elderly and immunocompromised individuals, underscoring the need for therapeutics that complement vaccines and reduce severe disease (Watson and Wilkinson, 2021; Williamson et al., 2020). Zoonosis can produce novel variants against which the population has limited or no preexisting immunity, and cause pandemics. Since new vaccines take time to develop, and often have reduced efficacy in more vulnerable individuals, broadly effective drugs will be essential for managing emerging infectious diseases. Current drugs targeting respiratory viruses are virus-specific and act by inhibiting viral enzymes or proteins (Beigel et al., 2020; Kanters et al., 2016). Because viruses mutate rapidly, antigenic drift can render such inhibitors ineffective (Kisoet al., 2004). Thus, broader antiviral strategies are needed. A promising approach is host-directed therapy, reducing the impact of viral mutations and opportunity of escape (Kaufmann et al., 2018; Saiz et al., 2018; Trimarco and Heaton, 2022).

It has previously been shown that host kinases play a role during infection. An example is the Mitogen-Activated Protein Kinase/Extracellular signal-Regulated Kinase (MAPK/ERK) pathway that is involved during influenza infection (Pleschka et al., 2001), and where inhibition have been demonstrated to also inhibit viral replication (Schräder et al., 2018). For the present context, Lymphocyte Tyrosine Kinase (LTK), the first known ER-resident receptor tyrosine kinase, regulates ER-to Golgi-trafficking, interacts with and phosphorylates Sec12 (Centonze et al., 2019), and controls ER exit site (ERES) biogenesis (Barlowe and Schekman, 1993). As such, LTK is important for COPII-dependent trafficking from ER to the Golgi, so drugs interfering with the proteostasis and secretion of vesicles could potentially limit virus replication in host cells. While COPII-dependent trafficking has not been established for influenza budding, models propose formation of liquid organelles at ERES (Alenquer et al., 2019). Similarly, coronaviruses have been proposed to depend on transport from ERES (Saraste and Prydz, 2021). We know from healthy and malignant plasma cells that inhibiting LTK prevents secretion of antibodies (Våtsveen et al., 2025). Given LTK’s role in protein transport and ERES biogenesis upstream of viral budding, we here hypothesized that LTK inhibition could reduce virion production in infected cells. Accumulation of viral proteins in the ER should induce cellular stress and trigger apoptosis of infected cells.

LTK is a paralogue of anaplastic lymphoma kinase (ALK), with ∼80% identity in their kinase domains (De Munck et al., 2021). ALK inhibitors are in clinical use for cancers with ALK translocations (Mastrantoni et al., 2024; Zhao et al., 2024), and we here therefore tested repurposed ALK inhibitors both for their ability to reduce viral secretion *in vitro* and for therapeutic efficacy in mice infected with influenza or SARS-CoV-2.

Relevant for respiratory viral infections, whereas LTK expression is highly abundant in human lung tissues, there is only sporadic expression of ALK in human lung tissues (Fig. 1A). The same dominance of LTK expression is also found in small intestine, which would be relevant for gastro-intestinal viruses (Fig. 1B) (Monaco et al., 2019). Among the highest expressors of LTK in human lungs we find ciliated epithelial cells along with endothelial cells (Supplemental Figure 1A). Ciliated epithelial cells are important for influenza replication (Roach et al., 2024). Since we were planning to evaluate LTK inhibitors in mice, we also examined the murine expression of LTK and ALK. We detected LTK in lung, bronchus, spleen and colon (Supplemental figure 1B), while ALK expression was only found in kidneys (Supplemental figure 1C) (Moreno et al., 2022). These expression patterns suggested that LTK can be targeted in relevant human and mouse tissues with minimal inhibitory ALK effects. This is important since the absence of ALK in lung and respiratory airways means that we can selectively target LTK when applying known ALK inhibitors to mice *in vivo*. In addition, the LTK kinase domain is also conserved with homology between human and mouse (91%), African green monkey (Chlorocebus sabaeus, Vero E6 cell line, 98%) and dog (Canis lupus, MDCK cell line, 81%), which is important for *in vitro* assays (Supplemental Fig. 2).

**Fig. 1:**
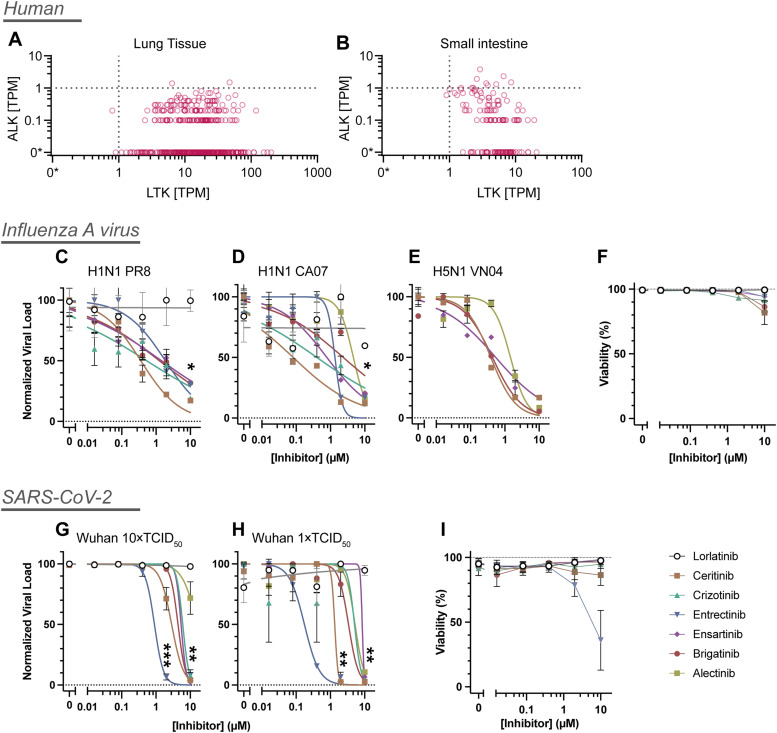
***In vitro* viral inhibition of Influenza A viruses and SARS-CoV-2. A**) Tissue wide gene expression data of LTK and ALK in human lung tissue. **B**) Tissue wide gene expression data of LTK and ALK in human small intestine. Data from the Genotype-Tissue Expression (GTEx) project (https://www.gtexportal.org). **C-I**) Monolayer cultures of MDCK cells (1 × 10^5^ cells/well) (C-F) or VeroE6 cells (1 × 10^4^ cells/well) (G-I) were seeded in 96-well plates and incubated with five-fold serially diluted LTK inhibitors in duplicates (Influenza) or triplicates (SARS-CoV-2), then infected with the indicated viruses. Infection was detected by the presence of influenza nucleoprotein or SARS-CoV-2 nucleocapsid in cells by ELISA after 50 h of incubation. Each point depicts the mean value from treatment with the indicated drug, with variation from technical replicates shown. **C**) Inhibition of A/Puerto Rico/8/1934 (H1N1) (PR8) (results from two merged separate experiments). **D**) Inhibition of A/California/07/2009 (H1N1) (CA07). **E**) Inhibition of A/Vietnam/1194/2004 (H5N1) (VN04). **F**) Cell viability of MDCK cultures exposed to inhibitors without virus, assessed by flow cytometry with viability dye (GhostDye510) after 50 h. **G**) Inhibition of SARS-CoV-2 at 10×TCID_50_viral dose. **H)** Inhibition of SARS-CoV-2 at 1×TCID_50_ viral dose. **I**) Cell viability of VeroE6 cultures exposed to inhibitors in the absence of virus, assessed by flow cytometry with viability dye (GhostDye510) after 50 h. Statistics were performed by one-way ANOVA for each inhibitor dose as compared to lorlatinib. *p < 0.0332, **p < 0.0021,***p < 0.0002.

We screened the chosen inhibitors (ceritinib, crizotinib, entrectinib, ensartinib, brigatinib, alectinib, and the cyclic ALK inhibitor lorlatinib, which uses a different binding pocket and has lower inhibitory concentration for LTK, as a negative control (Johnson et al., 2014)) against SARS-CoV-2 and influenza viruses *in vitro*. This was done by allowing the virus to infect host cells in the presence of different inhibitors, leading to reduced viral replication and viral load. Three influenza strains were included: A/Puerto Rico/8/1934 (H1N1) (PR8) (Fig. 1C), A/California/07/2009 (H1N1) (CA07) (Fig. 1D), and reassorted A/Vietnam/1194/2004 (H5N1)×A/Puerto Rico/8/1934 (H1N1) (VN04) (Fig. 1E). All three viruses were susceptible to inhibition *in vitro*. Importantly, the drugs had no effect on cell viability of MDCK cells in the absence of virus (Fig. 1F). Ceritinib had the lowest IC_50_ against the H1N1 strains (0.08 µM and 0.36 µM, respectively) and H5N1 (0.39 µM) (Table 1). Crizotinib, tested only against the two H1N1 strains, was also active with an IC_50_ of 0.40–0.53 µM. Prevention of SARS-CoV-2 in VeroE6 cells showed that entrectinib and ceritinib were the most effective, and where entrectinib gave an IC_50_ of 0.9 µM against a high viral dose (10×TCID_50_, Fig. 1G) and 0.2 µM against a low dose (1×TCID_50_, Fig. 1H). Ceritinib treatment resulted in an IC_50_ of 2.9 µM against a high dose and 1.3 µM against a low virus dose. The other inhibitors only showed activity at the highest concentration tested. The drugs were also here displaying negligible cytotoxicity when evaluated alone in VeroE6 cells (Fig. 1I). The negative control, lorlatinib, showed no activity against neither influenza nor SARS-CoV-2, indicating that the observed effect was due to LTK inhibition.

**Table 1:** *In vitro* viral inhibition IC_50_-values against influenza virus and SARS-CoV-2.

Inhibitor/Drug	Influenza IC_50_ [µM]			SARS-CoV-2 IC_50_ [µM]	
	*H1N1* *PR8*	*H1N1 CA07*	*H5N1* *RG14*	*1×TCID_50_*	*10×TCID_50_*
Lorlatinib	>10	>10	>10	>10	>10
Ceritinib	0.36	0.08	0.39	1.3	2.9
Crizotinib	0.53	0.40	—	5.0	6.0
Entrectinib	1.9	1.4	—	0.2	0.9
Ensartinib	1.4	0.78	0.54	8.5	5.4
Brigatinib	1.3	2.4	0.43	3.4	4.4
Alectinib	—	4.6	1.4	5.2	16.1

— = Not tested.

Based on *in vitro* screening, we proceeded to evaluate top candidates for protection against viral challenges in mice (Fig. 2A). First, as expected, neither drug caused signs of reduced health in the absence of virus, as indicated by weight (Fig. 2B). We next evaluated ceritinib, crizotinib, and brigatinib for protective efficacy in mice receiving a lethal dose of influenza PR8 (5×LD_50_) (Fig. 2C). All mice lost ≥10% body weight, but crizotinib delayed symptom onset by about one day. Survival was 37.5% (3/8) with crizotinib, 12.5% (1/8) with brigatinib, whereas no mice in treated with ceritinib or vehicle survived (Fig. 2D). We next challenged mice with an influenza CA07 virus (1×LD_50_), and evaluated ceritinib, crizotinib, entrectinib, and ensartinib for protective efficacy, with oseltamivir as a positive control. All inhibitor-treated groups lost weight at similar rates to vehicle during the first 7 days (Fig. 2E), whereas oseltamivir-treated mice had slightly reduced weight loss. Survival did not differ significantly between inhibitor-treated and vehicle groups (Fig. 2F), although surviving mice given inhibitors recovered faster than surviving vehicle controls. Together with the PR8 data, these results highlight crizotinib as the most promising candidate as it delayed symptom onset following PR8 infection and promoted faster recovery after the CA07 viral challenge.

**Fig. 2:**
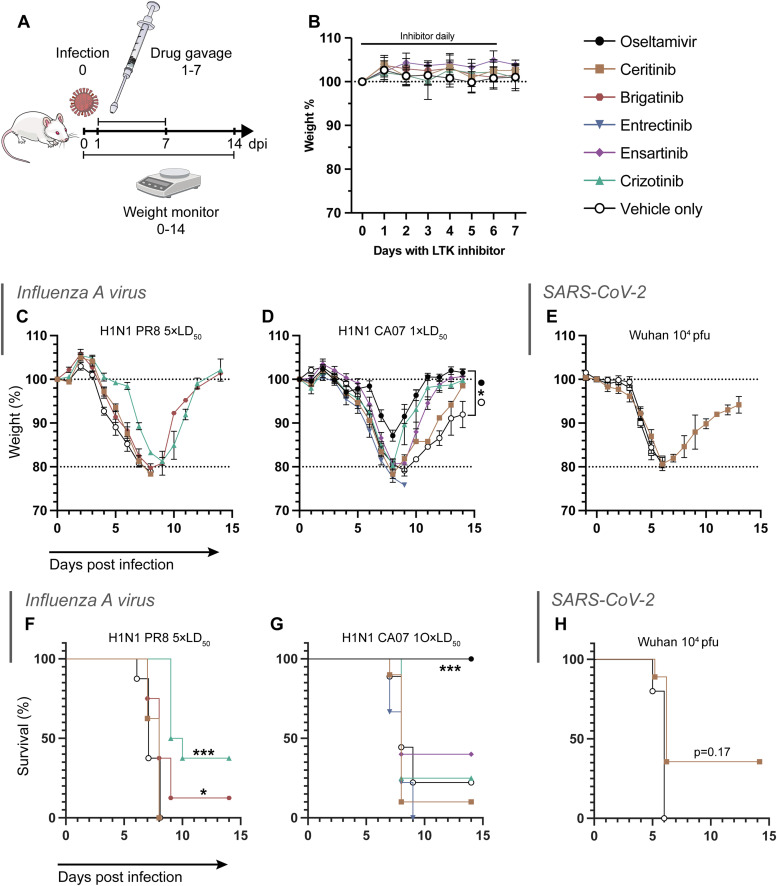
***In vivo* infection of mice treated with LTK inhibitors. A**) Schematic overview of experiment in mice. **B**) Tolerability assessment: female BALB/c mice were treated with LTK inhibitors (200 µl of 2.5 mg/ml, prepared in 5% DMSO, 30% PEG400, 65% H_2_O) by oral gavage daily for seven consecutive days, and body weight monitored. **C—H)** Mice were infected intranasally with the indicated viruses on day 0 (influenza: anesthetized with ZRF cocktail i.p.; SARS-CoV-2: anesthetized by isoflurane inhalation) and treated with inhibitors by daily oral gavage as above from day 1 until day 7. **C**) Weight following a 5×LD_50_ viral dose of influenza virus PR8 (n = 7–8 mice/group). **D)** Survival curve for B, with 20% weight loss as the humane endpoint. **E**) Weight following a 1×LD_50_ viral dose of influenza CA07 (n = 8–10 mice/group). **F)** Survival curve for D, with 20% weight loss as the humane endpoint. **G**) Weight following a10^4^ pfu dose of SARS-CoV-2 virus (K18-hACE2 transgenic mice on C57BL/6×BALB/c background) (n = 8–10 mice/group). **H)** Survival curve for F, with 20% weight loss as the humane endpoint. One-way ANOVA was evaluated for each day between days 10–14 as compared to vehicle only. Significance for survival curves were determined by Gehan-Breslow-Wilcoxon test for each inhibitor compared to vehicle only. *p < 0.0332, **p < 0.0021, ***p < 0.0002.

To also evaluate protective efficacy against a viral challenge with SARS-CoV-2, we decided to use ceritinib based on the combined results from the *in vitro* data against SARS-CoV-2 and influenza viruses. Early weight loss was similar between ceritinib‑treated and control mice (Fig. 2G). Despite this, 3/9 ceritinib‑treated mice survived versus 0/10 treated with vehicle (Fig. 2H). From this experiment we also evaluated lung tissues by Hematoxylin and Eosin staining (H&E), and found reactive epithelium and debris in bronchi. There were no clear differences at this early timepoint between mice treated with vehicle or ceritinib (day 5 of virus challenge). (Supplemental figure 3).

Overall, ceritinib provided some protection following a lethal viral challenge with SARS-CoV-2. On the other hand, crizotinib relieved disease burden after viral challenges with both of the tested H1N1 strains, while also being effective against influenza *in vitro*.

The present work explored the potential of repurposing ALK inhibitors for treatment of viral infection in a host-targeted therapeutics strategy by inhibiting LTK. The link between LTK and protein trafficking with an ERES association suggests that it could be used to specifically inhibit viral replication. The availability of such a broad-spectrum antiviral could help the fight against emerging potentially pandemic viruses where no vaccines currently exist.

Developing antiviral drugs for pandemic use is difficult because most drugs target a narrow range of viruses, and can be rapidly undermined by resistance. From a translational perspective, host-directed therapies by LTK-directed antivirals may supplement current virus-directed therapeutics. We know from before that resistance to protease inhibitors can be conferred by mutations in SARS-CoV-2 main protease and that the benefits of remdesivir is variable (Amani and Amani, 2023), with reports of resistance arising during persistent infection (Gandhi et al., 2022). Despite evidence of any direct interactions between LTK and viral proteins, ERES biogenesis is suggested to be involved in both influenza and coronavirus replication (Alenquer et al., 2019; Fung and Liu, 2014; Saraste and Prydz, 2021) and inhibition of LTK limit ER-to-Golgi trafficking (Centonze et al., 2019). By focusing on host ER trafficking nodes required for virion biogenesis, LTK inhibition could provide broad-spectrum durability across influenza and coronaviruses, complementing vaccines and virus-directed drugs. The observed cross-inhibition by multiple clinically approved ALK inhibitors facilitates rapid repurposing, supported by established pharmacology and safety data. In addition to the aforementioned MAPK pathway inhibitors, other repurposed kinase inhibitors investigated for potential antiviral properties include other receptor tyrosine kinases (Kumar et al., 2011), and protein kinase B (Akt) (Denisova et al., 2014).

In conclusion, repurposed ALK inhibitors targeting LTK represent a potential new class of antivirals with effects against multiple virus families in a host-directed fashion. The present work demonstrates feasibility for the strategy since there is an improvement of protection after treatment of mice with LTK inhibiting ALK inhibitors as compared to controls. However, further preclinical research is needed to refine delivery conditions and fully assess the potential of these drugs.

## Author contributions

E.T., L.M., and G.G. designed the study. E.T, T.K.V, M.R. and G.G. performed experiments and analyzed data. D.N. contributed to experimental design and interpretation of results. E.T., T.K.V., L.M. and G.G. wrote the paper, but all authors commented and edited the work.

## Author statement

No AI was used for preparation of the manuscript. This also includes text editing.

## Declaration of competing interest

GG, LM and HF are inventors on a patent application submitted via the TTO Inven2 on the repurposing of ALK inhibitors for use against viral infections.

## Appendix Supplementary materials

Image, application 1
