# Supplementary material for: Repurposing ALK inhibitors as influenza and corona virus antivirals targeting lymphocyte tyrosine kinase (LTK)
Source: Virus Res. 2026 Jun 9;370:199759. doi: 10.1016/j.virusres.2026.199759 (PMC13279203; doi:10.1016/j.virusres.2026.199759)
Supplement: Supplementary file 1 [file mmc1.docx]

**Supplemental information:**

**Repurposing ALK Inhibitors as Broad-Spectrum Antivirals Targeting Lymphocyte Tyrosine Kinase (LTK)**

Elias Tjärnhage^1^, Thea Kristin Våtsveen^1-4^, Melinda Raki^5^, David Nemazee^6^, Hesso Farhan^7,8^, Ludvig Munthe^1-4^ *, and Gunnveig Grødeland^1,2,9^*

^1^Institute of Clinical Medicine, University of Oslo, Oslo, Norway

^2^Department of Immunology, Oslo University Hospital, Oslo, Norway

^3^KG Jebsen Centre for B cell malignancy, Institute of Clinical medicine, University of Oslo, Oslo, Norway.

^4^PRIMA, Precision Immunotherapy Alliance, University of Oslo, Oslo, Norway

^5^Department of Pathology, Oslo University Hospital, Oslo, Norway

^6^The Scripps Research Institute, La Jolla, California, USA.

^7^Institute of Basic Medical Sciences, University of Oslo, Oslo, Norway

^8^ Institute of Pathophysiology, Medical University of Innsbruck, Innsbruck, Austria

^9^Centre for Pandemics and One-Health Research, Institute of Health and Society, University of Oslo

*Shared senior authorship

Corresponding Authors: Elias Tjärnhage ([Elias.tjarnhage@medisin.uio.no](mailto:Elias.tjarnhage@medisin.uio.no)) and Gunnveig Grødeland ([Gunnveig.grodeland@medisin.uio.no](mailto:Gunnveig.grodeland@medisin.uio.no))

**MATERIAL and METHODS**

***LTK/ALK gene expression analysis***

Transcriptomics data were accessed through EMBL-EBI expression atlas (<https://www.ebi.ac.uk/gxa>) and single cell expression atlas (<https://www.ebi.ac.uk/gxa/sc/home>), as well as the Protein Atlas Genotype-Tissue Expression (GTEx) project of human post mortem tissues (<https://www.proteinatlas.org/>) for human LTK (ENSG00000062524) and ALK (ENSG00000171094), or for mouse equivalents (LTK, ENSMUSG00000027297, ALK ENSMUSG00000055471). Data from the published datasets were accessed (Habermann et al., 2020; Lonsdale et al., 2013; Monaco et al., 2019; Uhlén et al., 2015; Ziegler et al., 2020). LTK expression in Vero E6 cells was confirmed by RT-qPCR using the following Taqman probes for human kinases: ALK Hs00608284_m1, LTK Hs01587782_g1 and PGK1 Hs00943178_g1 on isolated mRNA (data not shown).

***ALK/LTK inhibitors***

All ALK/LTK inhibitors were purchased from Selleckchem (Selleck Chemicals GmbH, Germany): Lorlatinib (Cat No. S7536), Ceritinib (Cat No. S7083), Crizotinib (Cat No. S1068), Entrectinib (Cat No. S7998), Ensartinib (Cat No. S2934), Brigatinib (Cat No. S8229), and Alectinib (Cat No. S2762).

***In vitro viral inhibition***

*Influenza:* 1×10^5^ Madin Darby Canine Kidney (MDCK) cells were seeded in 96-well plates (Cat No. Costar 3595, Corning Incorporated, NY, USA) in DMEM (Cat No. 12077549, ThermoFisher, NJ, USA) with 2% Fetal Bovine Serum (FBS) (Cat No. 10270-106, ThermoFisher). Five-fold dilution series of ALK/LTK inhibitor compounds were then added in duplicates followed by 100×TCID_50_ influenza virus. The cells were then incubated for 50 hours at 37°C and 5% CO_2_ in a humidified incubator. Included strains were: influenza A/Puerto Rico/8/1934(H1N1) (PR8), influenza A/California/09/2007(H1N1) (CA07), and NIBRG14 [PR8 viral backbone reassorted with surface proteins from influenza A/Vietnam/1194/2004(H5N1) (VN04)]. After incubation, the cells were washed with PBS and fixed with ice-cold 80% acetone in PBS for 10 minutes before left to dry.

*SARS-CoV-2:* 1×10^4^ Vero E6 cells were seeded per well into 96-well tissue culture plates (Cat No. Costar 3595, Corning Incorporated) in DMEM (Cat No. 12077549, ThermoFisher) with 2% FBS (Cat No. 10270-106, ThermoFisher). Five-fold dilution series of inhibitor compounds were added in triplicates, followed by incubation with either 1×TCID_50_ or 10×TCID_50_ SARS-CoV-2 virus (2019nCoV/Italy, INMI1: 008V-03893 European Virus Archive). Following incubation at 37°C in a 5% CO_2_ humidified atmosphere for 50 hours, cells were washed with PBS and fixed for 30 minutes in ice-cold 80% acetone.

Infection was detected using an ELISA assay, and where influenza cellular infection was detected by incubation overnight at 4°C with biotinylated anti-nucleoprotein IgG (clone: H16-10L-4RS, produced in-house) followed by Streptavidin-alkaline phosphatase (Cat No. 7105-04, Southern Biotech, AL, USA) for 60 minutes at room temperature, before development of plates using 1 mg/ml phosphatase substrate (P4744-10G, Sigma Aldrich). Cellular infection with SARS-CoV-2 was detected in the same manner as influenza, except that an anti-nucleocapsid rabbit IgG (Cat No. 40143-R004, SinoBiological, China) and an HRP-conjugated goat anti-rabbit IgG mAb (Cat No. SSA003, Sino Biological) was used, and then development was by TMB substrate buffer (Cat No. sc-286967, Santa Cruz Biotechnology. TX, USA) and 1M HCl. Absorbance from all plates were read with an EnVision 2104 Multilabel Reader (Perkin Elmer).

***In vitro cytotoxicity analysis***

MDCK cells and VeroE6 were seeded as described for the *in vitro* viral inhibition, and 5-fold dilutions of LTK inhibitors were added in triplicates. Following incubation at 37°C in a 5% CO_2_ humidified atmosphere for 50 hours, cells in the cell culture supernatant were transferred to a 96-well V-bottom plate before any remaining adherent cells were detached by trypsination and transferred to the same plate. Cells were then washed and stained with viability dye (1:400 GhostDye510. Cat. No. 13-0870-T500, Tonbo Bioscience) and data was acquired on an Attune NxT flow cytometer (Thermofisher).

***In vivo inhibitor preparation and viral challenge***

Powdered ALK/LTK inhibitors were dissolved in DMSO at 50 mg/ml, with heating of the mixture at 42°C if necessary. Each drug (5% final DMSO concentration) was first mixed with PEG400 (Cat No. 06855, Merck) (30% final concentration), followed by addition of MilliQ H_2_O (65% final concentration), and mixed until a homogenous solution was obtained (2.5 mg/ml working concentration drug). Each dose was prepared fresh from the 50 mg/ml stock solution each morning. Unfortunately, the solubility of Ceritinib in DMSO was less than required for the desired 50 mg/ml stock. Therefore, a well dispersed suspension of 50 mg/ml in DMSO was used as stock instead.

*Influenza challenges:* Female BALB/c mice were anesthetized by intra peritoneal (i.p.) injection of ZRF cocktail [Zolazepam (3.3 mg/ml), Tiletamine (3.3 m/ml), Xylazine (0.45 mg/ml), Fentanyl (2.6 µg/ml)] at 10 µl/gram body weight, and challenged with a 5×LD_50_ dose of virus in 20 µl intranasally (i.n.) of influenza PR8 or 2×LD_50_ of influenza CA07. Starting the day after viral challenge, mice were given 200 µl of 2.5 mg/ml inhibitor orally daily for 7 days post infection.

*SARS-CoV-2 challenge*: A mix of male and female K18-hACE2^het^C57/BL6×BALB/c crossed mice were confirmed genotyped for hACE2 expression before starting experiments. The mice were anesthetized by isoflurane inhalation and then given an i.n. 50 µl virus suspension (10^4^ pfu per mouse). Starting the day after viral challenge, 200 µl Ceritinib were given orally at 0.14 mg/ml daily for 7 days post infection.

*In vivo tolerability of LTK inhibitors*: Female BALB/c were administered LTK inhibitors orally as described above for 7 consecutive days without receiving virus and the mice were weighed daily.

***Animal housing***

All BALB/c mice were purchased 6-8 weeks old from Janvier labs (BALB/cAnNRj, Janvier Labs, France) and housed at Oslo University Hospital, Department of Comparative medicine, Oslo, Norway, in a BSL2 facility approved for work with infectious material. All experiments with influenza virus were approved by the Norwegian Animal Research Authority. The K18-hACE2 mice were purchased 6-8 weeks old from Jackson Laboratories (B6.Cg-Tg(K18-ACE2)2Prlmn/J, #034860, Jackson Laboratories, ME, USA) and then housed and bred in-house at The Scripps Research Institute, La Jolla, CA, USA. Before the SARS-CoV-2 challenge, mice were transferred to a BSL3 approved vivarium at The Scripps Research Institute. All animal experiments with SARS-CoV-2 were approved by the AALAS.

***Lung histopathology***

Lungs were dissected from representative mice (median weight loss per group) at day 5 after viral challenge (SARS-CoV-2, 2019nCoV/Italy), and immediately rinsed in PBS before fixation and storage in formalin. Representative mice were chosen based on median weight loss on the day prior to lung dissection. The lungs were then paraffin-embedded, sliced, and stained with hematoxylin and eosin (H&E). Finally, the lungs were evaluated for changes in the airways and the parenchyma.

***Statistical Analysis***

All statistical analysis was performed in Graphpad Prism v10. In vitro inhibition analysis was done by comparing each inhibitor against the negative control (lorlatinib) at each drug concentration by one-way ANOVA (Brown-Forsythe and Welch test with Dunnett’s T3 mutliple comparison). *In vivo* body weight analysis was done by day-wise comparison of each inhibitor group to the vehicle only group using one-way ANOVA from day 10-14 (Kruskall-Wallis test with Dunn’s multiple comparison). Significance was indicated if at least three days were significant of the days tested. In vivo survival analysis was done by comparing each inhibitor group to the vehicle only group using the Gehan-Breslow-Wilcoxon test.

**Supplemental figures:**


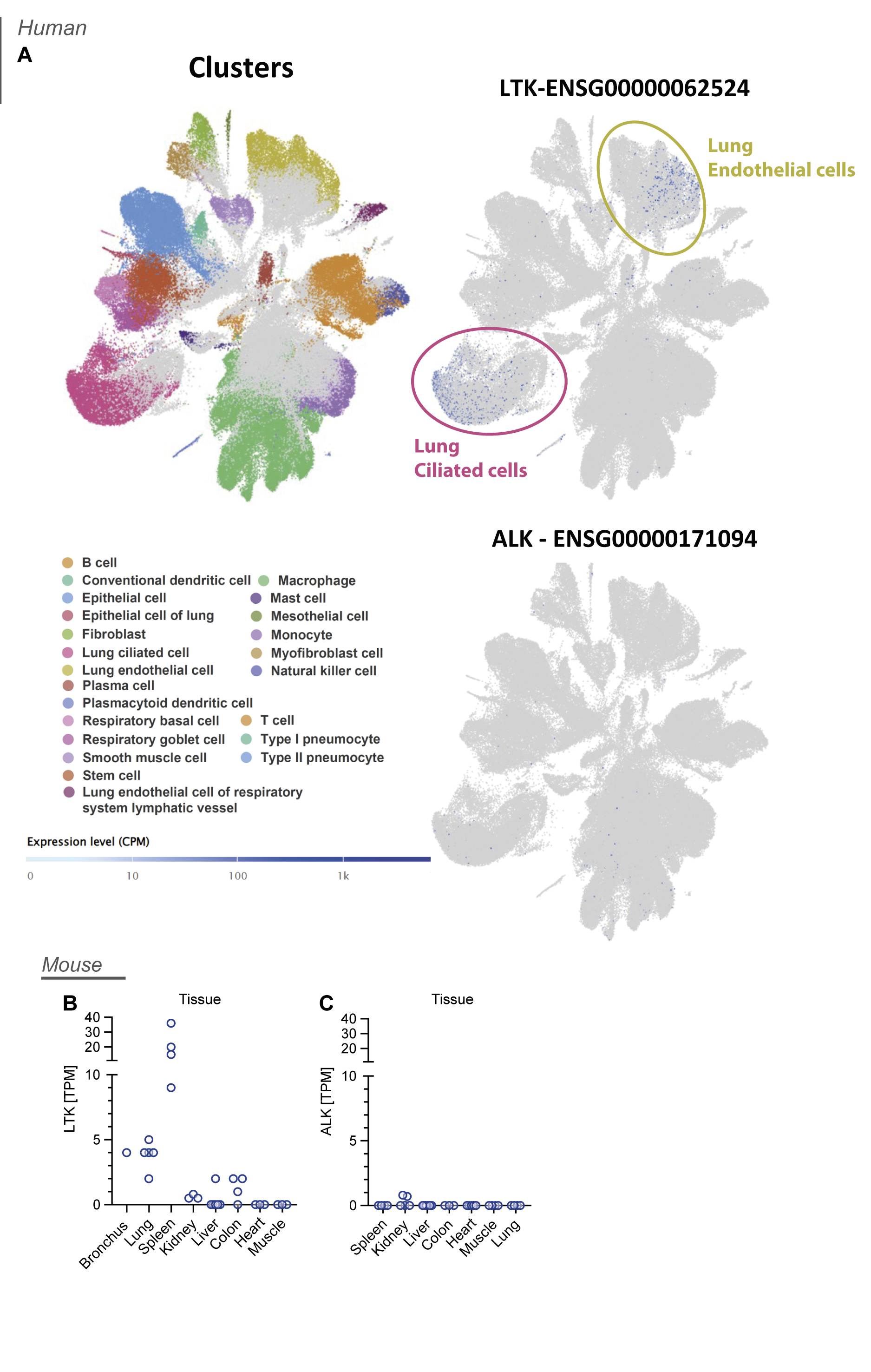


**Supplemental figure 1: LTK expression in lung and nasal mucosa cell types.** **A**) Single cell expression of LTK and ALK. Human scRNASeq data of lung cells clusters are shown (top left) with expression of LTK (top right and ALK (lower right). **B-C)** Expression of C) LTK and D) ALK in different mouse tissues. Data from the Expression Atlas (https://www.ebi.ac.uk/gxa/home)(Moreno et al., 2022).


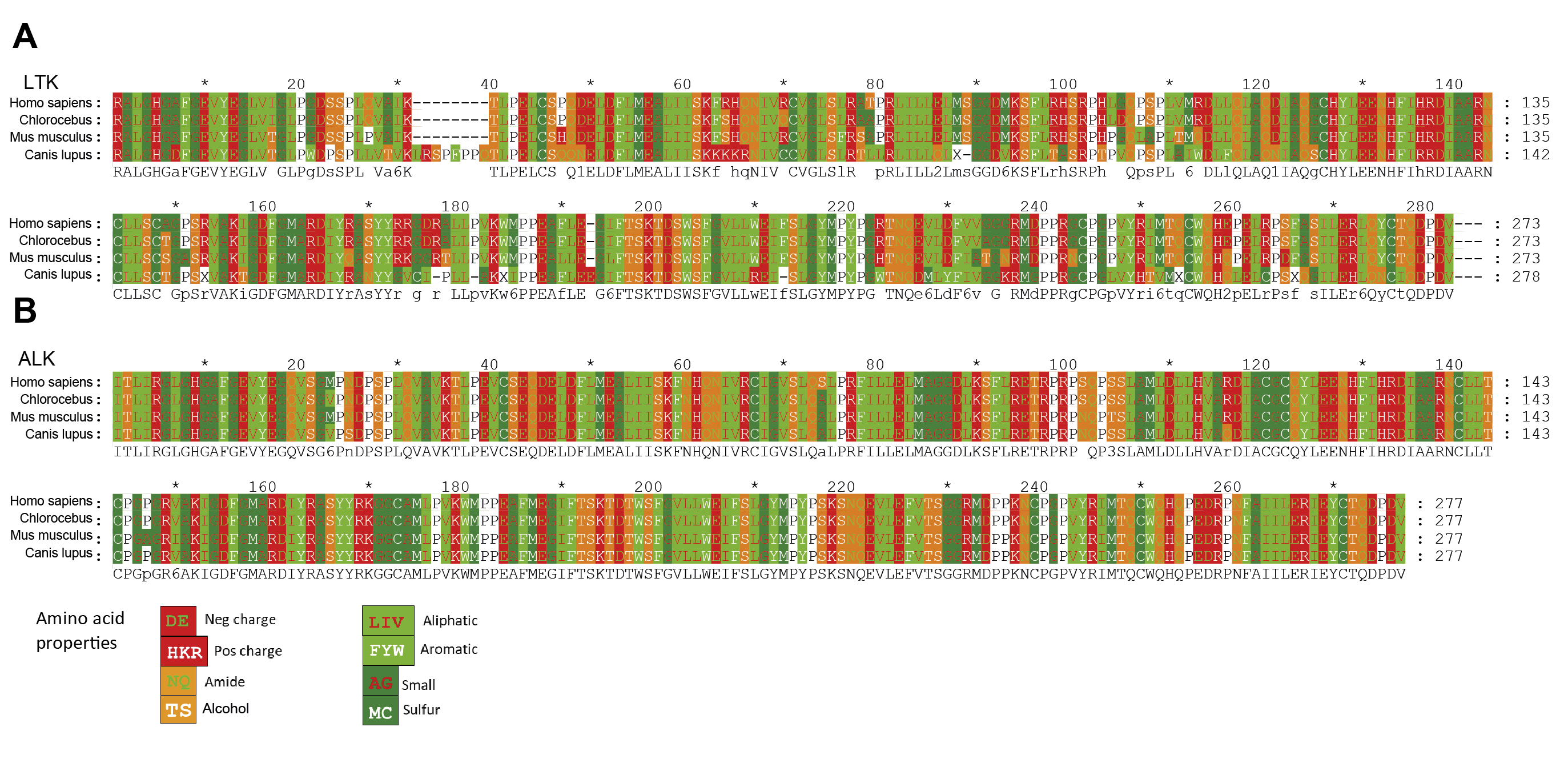


**Supplementary figure 2: Sequence alignment of (A) LTK and (B) ALK homologs between species.** Alignments of reference sequences of human (Homo Sapiens), monkey (Chlorocebus sabaeus), mouse (Mus musculus), and dog (Canis lupus familiaris) were performed using ClustalO (Uniprot.org) and presented with GeneDoc, showing amino acids with similar physiochemical properties (see indicated key). Kinase domains are shown from the respective NCBI reference protein sequences, <https://www.ncbi.nlm.nih.gov/protein>. LTK: human XP_011519859.1, Chlorocebus sabaeus XP_037866211.1, mouse NP_032549.2, Canis lupus XP_038297733.1. ALK: human XP_054197220.1, Chlorocebus sabaeus XP_007969246.2 , Mus musculus XP_036016161.1, Canis lupus P0DV84.

LTK kinase domain is conserved with homology between human and mouse (91%), African green monkey (Chlorocebus sabaeus, Vero E6 cell line, 98%) and dog (Canis lupus, MDCK cell line, 81%).


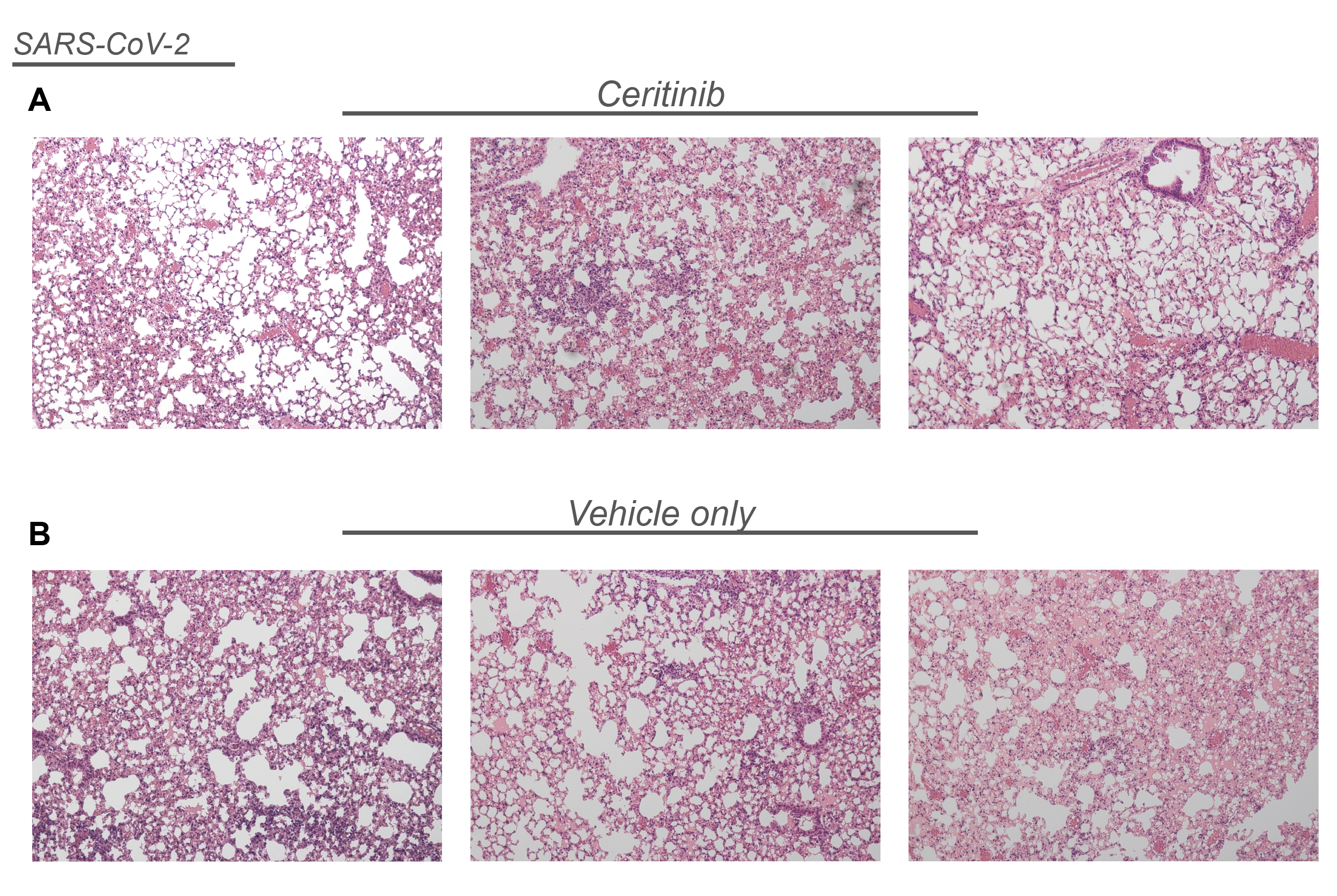


**Supplemental figure 3. HE-staining of lungs after SARS-CoV-2 challenge.** Lungs from 3 representative mice (chosen based on median weight) per group (Figure 3C) were harvested 5 days post a viral challenge with SARS-CoV-2, and stained for H&E. **A)** Lungs from ceritinib treated mice. **B)** Lungs from non-treated mice receiving vehicle only.
